# Design and Performance of a 1 ms High-Speed Vision Chip with 3D-Stacked 140 GOPS Column-Parallel PEs †

**DOI:** 10.3390/s18051313

**Published:** 2018-04-24

**Authors:** Atsushi Nose, Tomohiro Yamazaki, Hironobu Katayama, Shuji Uehara, Masatsugu Kobayashi, Sayaka Shida, Masaki Odahara, Kenichi Takamiya, Shizunori Matsumoto, Leo Miyashita, Yoshihiro Watanabe, Takashi Izawa, Yoshinori Muramatsu, Yoshikazu Nitta, Masatoshi Ishikawa

**Affiliations:** 1 Sony Semiconductor Solutions, Japan, 4-14-1 Asahi-cho, Atsugi-shi, Kanagawa 243-0014, Japan; Tomohiro.X.Yamazaki@sony.com (T.Y.); Hironobu.Katayama@sony.com (H.K.); Shuji.Uehara@sony.com (S.U.); Masatsugu.Kobayashi@sony.com (M.K.); Sayaka.Shida@sony.com (S.S.); Takashi.Izawa@sony.com (T.I.); Yoshinori.Muramatsu@sony.com (Y.M.) Yoshikazu.Nitta@sony.com (Y.N.); 2 Sony LSI Design, Japan, 4-16-1 Okada, Atsugi-shi, Kanagawa 243-0021, Japan; Masaki.Odahara@sony.com (M.O.); Kenichi.Takamiya@sony.com (K.T.); Shizunori.Matsumoto@sony.com (S.M.); 3 Graduate School of Information Science and Technology, The University of Tokyo, 7 Chome-3-1 Hongo, Bunkyō, Tokyo 113-8654, Japan; Leo_Miyashita@ipc.i.u-tokyo.ac.jp (L.M.); Yoshihiro_Watanabe@ipc.i.u-tokyo.ac.jp (Y.W.); Masatoshi_Ishikawa@ipc.i.u-tokyo.ac.jp (M.I.)

**Keywords:** vision chip, high-speed image sensor, detection, tracking

## Abstract

We have developed a high-speed vision chip using 3D stacking technology to address the increasing demand for high-speed vision chips in diverse applications. The chip comprises a 1/3.2-inch, 1.27 Mpixel, 500 fps (0.31 Mpixel, 1000 fps, 2 × 2 binning) vision chip with 3D-stacked column-parallel Analog-to-Digital Converters (ADCs) and 140 Giga Operation per Second (GOPS) programmable Single Instruction Multiple Data (SIMD) column-parallel PEs for new sensing applications. The 3D-stacked structure and column parallel processing architecture achieve high sensitivity, high resolution, and high-accuracy object positioning.

## 1. Introduction

High-speed vision systems that combine high frame-rate imaging and highly parallel signal processing enable extremely high-speed visual feedback to more rapidly control machines as compared to human-visual-recognition speeds. Such systems enable a reduction in circuit scale by using a fast and simple algorithm optimized for high frame-rate processing [1]. Previous works on such vision systems and chips [1,2,3,4] have yielded low imaging performance due to large matrix-based processing elements (PE) [1,2,3] and low functionality of the limited-purpose column-parallel PE architecture [4], constraining vision-chip applications. Dynamic Vision Sensors (DVSs) [5,6,7,8] also enable remarkably high-speed detection of intensity changes by using event-based operation. However, a large pixel size for event-based operations limits the spatial resolution and the external processor and memory for complex data reconstruction and recognition processing create a large-scale system.

CMOS Image Sensors (CISs) have rapidly replaced CCD image sensors in most commercial camera applications due to their superiority in power consumption and higher flexibility of signal readout architecture. CIS technology has improved its image quality along with shrinking pixel size, increasing sensor resolution, enabling sophisticated signal processing, and allowing new CIS architectures such as Back-Illuminated (BI)-CIS and imager plus logic stack technologies [9,10,11,12,13].

However, there has not been a vision chip realized that achieved high sensitivity, high resolution, and high-accuracy object positioning. This paper presents a 1/3.2-inch, 1.27 Mpixel, 500 fps (0.31 Mpixel, 1000 fps, 2 × 2 binning) vision chip with a 3D-stacked column parallel Analog-to-Digital Converter (ADC) [14] and 140 GOPS programmable SIMD column parallel PEs for diverse sensing applications [15]. The architecture of the parallel signal processor was developed as an image processing Digital Signal Processor (DSP) [16,17,18] and the technology of the processor is applied to the vision chip to realize high-speed sensing. The architecture of the parallel signal processor is like the column-based readout sensor architecture. 

This paper explains the details of the high-speed vision chip based on the IISW paper [19].

## 2. Materials and Methods 

**Figure 1:**
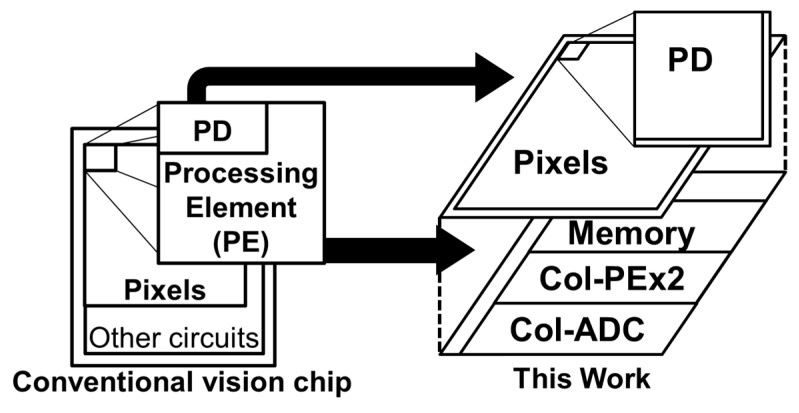
3D stack vision chip.

Our approach to the high-speed vision chip is to adopt a 3D-stacked structure. We use a stacked column ADC/PE structure to realize good imaging capability and two-column PEs with memory and a serial sensing processor to realize fast and flexible functionality. Our approach is shown in Figure 1. There are three benefits of the 3D stacked structure: (a) high resolution, (b) high sensitivity, and (c) high functionality. 

The 3D-stacked structure uses separate logic and pixel processes for implementing the two chips. The upper chip consists of only pixels. It can achieve high resolution and high sensitivity with back-illuminated pixels [20]. The lower chip, using an advanced logic process, contains large amounts of logic. The 3D stack structure realizes high imaging capability along with fast and flexible functionality.

**Figure 2:**
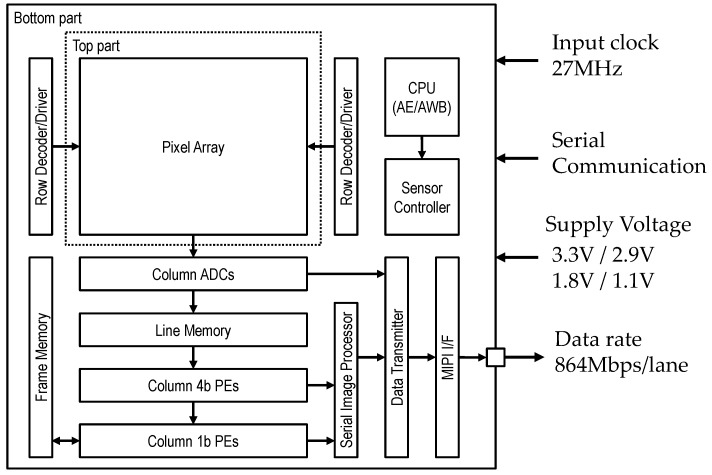
Block diagram of the chip.

The block diagram of the chip is shown in Figure 2. This sensor consists of the pixel array, the column parallel single-slope ADCs, the column parallel 4b/1b PEs, a 1b frame memory, a 4b/1b serial image processor, a 12b/10b/4b image data transmitter, MIPI interface, and a CPU connected with a sensor controller. The ADCs operate in 4b mode for high-speed vision sensing and in 12b/10b mode for viewing the output. The architecture, composed of column parallel ADCs, PEs, and frame memory, solves the constraint of the process-able high pixel count due to the number of PEs and enables 140 GOPS 4b inter-pixel and 1b inter-frame operations. The serial image processor aggregates the data from column PEs to realize various sensing functions. The image data transmitter embeds the results of vision sensing into the image data and outputs images. The speed of the MIPI interface is 864 Mbps/lane using 4 lanes. The CPU performs high-speed Auto Exposure (AE) and Auto White Balance (AWB) and feeds back to the sensor controller to improve the robustness of the imager to address lighting variations for full vision sensing.

**Figure 3:**
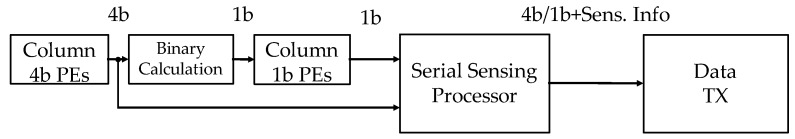
Data flow for sensing.

The high-speed vision chip realizes two sensing functions: (a) target detection using color or a condition of the brightness of each pixel, (b) target tracking using centroid movement of target object. The sensing functions are realized in the lower chip as a large logic function. The data flow of the chip for sensing is shown in Figure 3. There are three important blocks for sensing. The 4b column PEs perform pre-filtering for spatial processing to extract targets based on luminance and color information. The binary block in column 1b PEs binarizes pixels according to the binarizing parameter. The Column 1b PEs performs noise reduction for temporal processing. Two columns PEs perform column parallel processing that is suitable for column parallel data conversion. The 4b PEs and the 1b PEs execute parallel to serial data transfer, and output to a serial sensing processor. The Serial Sensing Processor aggregates 4b/1b data from column PEs to generate sensing output information. 

**Figure 4:**
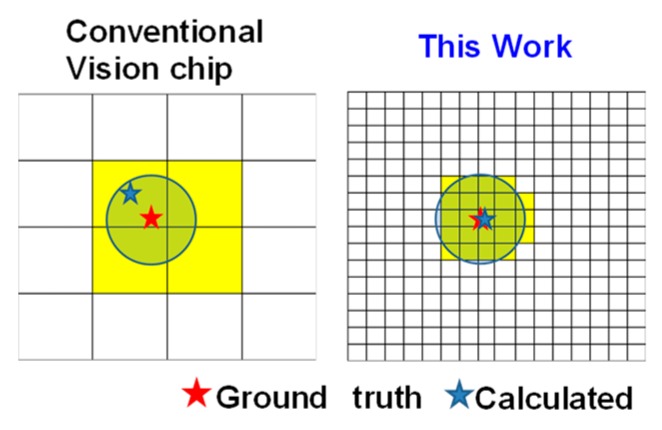
Improvement of sensing accuracy.

Low spatial resolution causes low accuracy in acquiring the target’s centroid. High resolution improves the accuracy of centroid position. The improvement of sensing accuracy is shown in Figure 4. Ground truth means the true position of the centroid.

**Figure 5:**
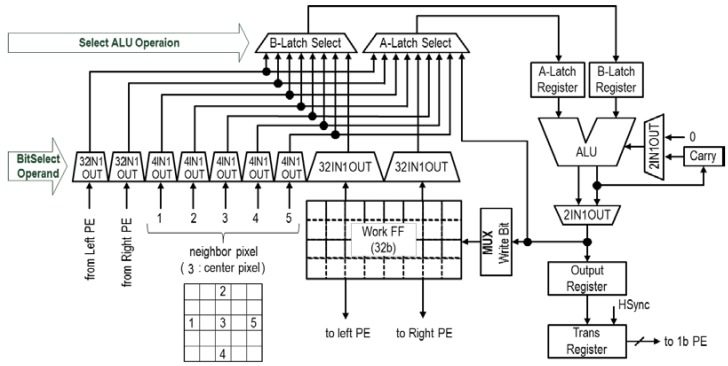
Schematic of the 4b PE.

The schematic diagram of the 4b programmable PE is shown in Figure 5. This circuit performs “1 ms (1000 frames per second)” high-speed 4b readout and processing. The first 4b column PEs perform spatial processing with the five-line memories. The 4b PE consists of a compact, bit-serial Arithmetic Logic Unit (ALU), 32 bits of local memory, an ALU operand selector, and an output register. This 4b PE can be controlled by eight instructions and the bit-serial ALU supports arithmetic operations, a shift operation, and logical operations. To achieve processing within only a few cycles, all selection signals to the PE are fixed in one instruction. The 4b PEs achieve 140 GOPS at a 108 MHz peak frequency because a 1b primitive operation takes one cycle and 1304 PE × 108 MHz = 140 GOPS. The PE inputs can be selected from three data sources; neighboring pixel data, processed data in the neighboring PEs, and data in its own work FF. The PE is connected to the neighboring left and right PEs. The PE receives horizontal data over two neighboring pixels, repeating the copy function using work FF. The PEs can also access vertical data in five line memories. The PE achieves various functions within only a few cycles using a carry register and ALU performing arithmetic operations. These data allow for flexible spatial operations.

**Figure 6:**
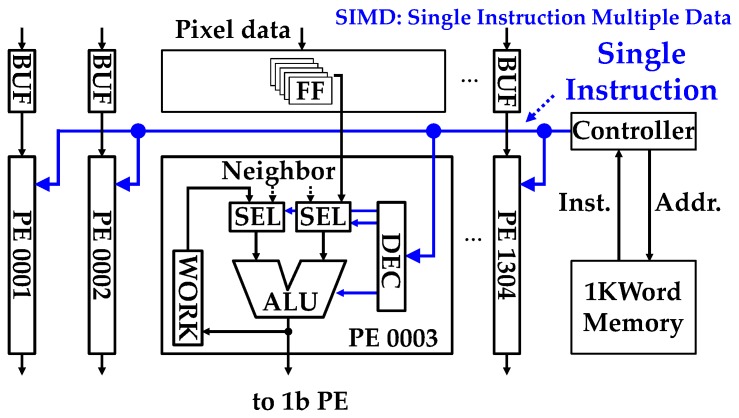
Column 4b PEs SIMD operation.

The schematic diagram of the 4b PE SIMD controller is shown in Figure 6. A 1000 word memory is implemented for instruction memory. The controller connects all 1304 4b PEs and reads out one instruction from the memory and issues it to all PEs in parallel. The controller executes an instruction each cycle with pipelined operation. All PEs can be operated simultaneously at 108 MHz. The controller can store a maximum of four different programs in 1000 word instruction memory. The programs can be configured simultaneously and an effective operation program can be selected for each frame or line. It enables spatial processing by color and dynamic program selection that adapts sensing operations to improve the accuracy of processing.

The basic operations and their steps of the 4b PEs are shown in Table 1. The basic operations are arithmetic operations, a shift operation, logical operations, as well as combinations of some instructions. The instructions have 55-bit length including ALU setting, carry in setting, selected input data, and selected output data. The operation code is stored by the 1000 word memory, and are executed sequentially. The processor includes a jump operation. These are reset horizontal sync timing, and carry out from a beginning code, or code specified by jump operation. These processor steps have different timings based on the number of bits of data.

These operations are fully pipelined. The vision chip can take a maximum of 2 µs as processing period of line.

**Figure 7:**
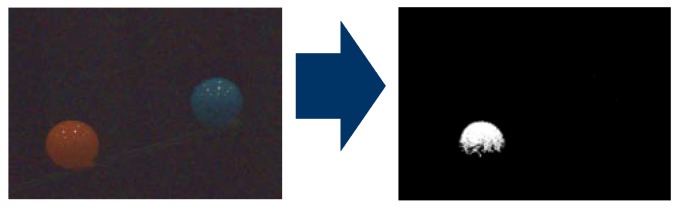
Red ball detection.

The 1b PEs is the second block in the sensing function. First, this block binarizes 4b input data from the 4b PEs. Each pixel is binarized with a threshold of the brightness or the color. The pixel within threshold is recognized as the target object. For example, the binary calculation image is shown in Figure 7. The left image is input data and the right image is binarized data. There are two balls, red and blue. In the image in this example, the red ball is detected. The threshold of binarization is color. The binarized block extracts only the red ball.

The color mode needs a specified target color (red, green, or blue) and four parameters, *Threshold_Min*, *Threshold_Max*, *Color1_Weight*, and *Color2_Weight*. Based on the settings, binarized data are given by the following conditions:

*Threshold_Min* ≤ *Target_Color* ≤ *Threshold_Max* (1)

*(1 + Color1_Weight)* × *Comparison_Color1* ≤ *Target_Color,* (2)

*(1 + Color2_Weight)* × *Comparison_Color2* ≤ *Target_Color*. (3)

*Target_Color* is each pixel value of specified color. Once the target color is selected, comparison colors are decided automatically. *Comparison_Color1* and *Comparison_Color2* are each pixel value of comparison colors. Based on threshold parameters (*Threshold_Max* and *Threshold_Min*), the binarized range of the target color is determined. Weighting parameters (*Color1_Weight*, *Color2_Weight*) are used for color gamut specification. A coefficient *Color1_Weight*/*Color2_Weight* is a ratio between comparison colors. Binarizing in comparison colors is performed by the equations, which use the ratio between target color value and comparison color value.

**Figure 8:**
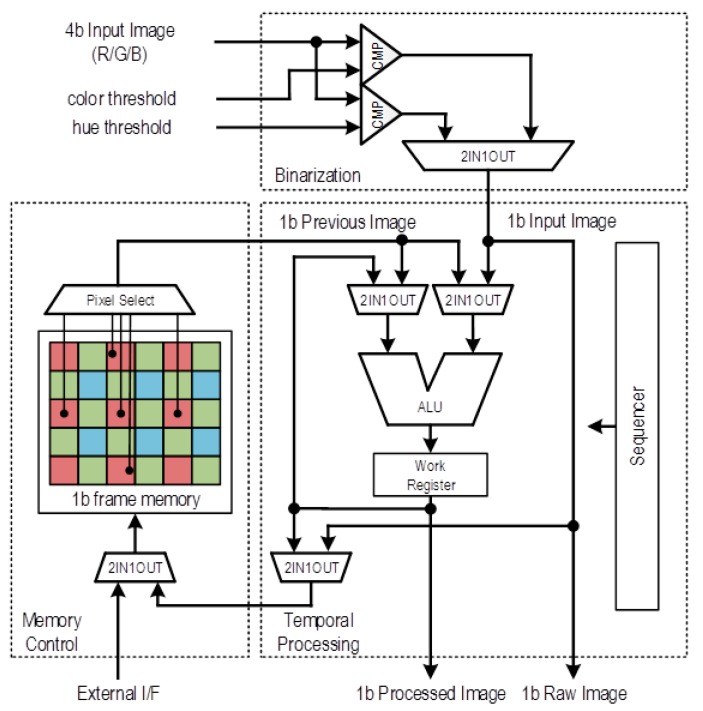
Schematic of the 1b PE with frame memory.

The schematic diagram of the 1b PE with frame memory is shown in Figure 8. The ALU in the 1b PE consists of primitive logic gates for 1b inter-frame operations such as self-window method [1], or inter-frame difference method to extract moving objects. The 1b PEs enable chroma-based binarization by thresholds for each color or hue threshold and target detection using the temporal variation between a current frame and the previous frame buffered in the 1b frame memory. 4b spatial processing such as smoothing filtering improves the accuracy of target extraction by reducing the spatial random noise. The AE function also helps binarization to adapt dynamic luminance change.

The functions of the 1b PE are shown in Table 2. The PE can perform three temporal functions: Self-Window method [21], Frame difference, and Background difference target extraction methods. 

**Figure 9:**
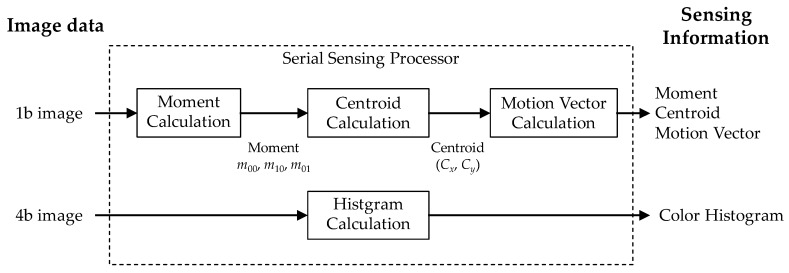
Block diagram of the serial image processor.

The block diagram of the serial image processor is shown in Figure 9. The sensing processor is a ladder circuit of two column parallel PEs and includes a target moment calculator, a centroid calculator, and a motion vector calculator. The processor aggregates 4b/1b data from the column PEs to generate sensing information. This data aggregation does not require parallel processing. We chose this serial architecture to achieve a small circuit size.

The serial processor calculates target information such as moment, centroid, and motion-vector with a maximum of six Regions of Interest (ROIs) and RGB histogram calculation to process the sensing information. The size (height and width) of ROI window is not configured automatically and can be configured from external system via writing sensor’s registers. The accuracy of moments suffers from spatial noise. 4-bit filtering, such as smoothing, can improve the accuracy of moments by temporal noise reduction. The target’s 0th moment *m*_00_, and 1st moments *m*_10_, *m*_01_ are given by the following equations:

(4) $$m_{00}=\sum_{x}\sum_{y}I_{bin},$$

(5) $$m_{10}=\sum_{x}\sum_{y}xI_{bin},$$

(6) $$m_{01}=\sum_{x}\sum_{y}yI_{bin}.$$

Here, *x* and *y* are the horizontal and vertical pixel positions, respectively, and *I_bin_* is the binarized pixel value (0 or 1) in the 1b PE. The centroid (*C_x_*, *C_y_*) is given by the following equations using the moments:

(7) $$C_{x}=\frac{m_{10}}{m_{00}},$$

(8) $$C_{y}=\frac{m_{01}}{m_{00}}.$$

**Figure 10:**
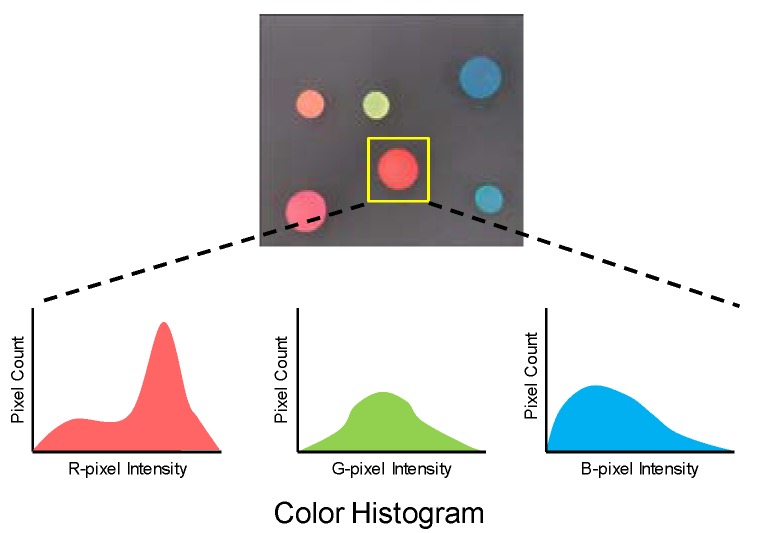
RGB histogram calculation.

RGB histogram calculation is shown in Figure 10. The serial processor calculates an RGB histogram for each ROI. The RGB histogram has 16 bins as the pixel is 4 bits in length.

**Figure 11:**
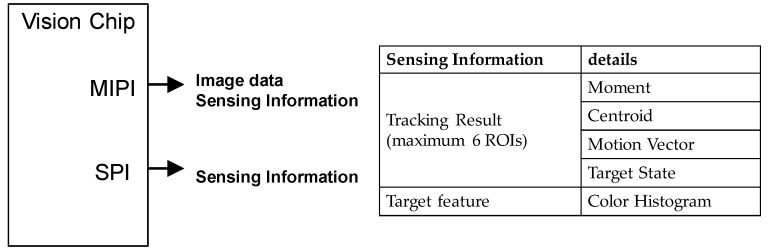
Output information.

Sensing information such as the moments and centroids is embedded into the image data, and transmitted to the output interface. Output information is shown in Figure 11. A high-speed vision system includes the vision chip, flash memory for program code, and an external controller. The external controller is connected to the chip and gets sensing information from the chip using MIPI or SPI. The MIPI interface transfers image data and sensing information is transferred by the SPI interface. The external controller such as a micro controller calculates the threshold parameter for binarizing using the RGB histogram, calculates the search area for detection using target state, and calculates ROI window size for tracking moment and centroid. The chip outputs sensing information for each frame (2 ms@500 fps, 1 ms@1000 fps). 

In this experiment, the MIPI interface output from the vision chip is converted to USB interface and processed by a Windows PC. In the processing on the PC side, vision chip control (designation of ROI window size, from detection to tracking switching) is performed using sensing information redundant in MIPI interface data. Only the UI for displaying the experiment results is executed.

## 3. Results

The specifications of the vision chip are summarized in Table 3. The upper chip is fabricated using a 90 nm, 1P4M MOS process. The lower chip is fabricated using a 40 nm 1P7M logic process. The chip performance achieves 1.27 Mpixel, 500 fps operation at 4b resolution, and 0.31 Mpixel, 1000 fps at 4b resolution. Power dissipation of one PE is 0.23 mW/GOPS. Power consumption is 230 mW at 1.27 Mpixel 12b 60 fps without sensing or 363 mW at 0.31 Mpixel 4b 1000 fps with sensing. The column PEs consumes only 32 mW. Processor count is one CPU for sensor control and 1304 column parallel PEs. Instruction cycle is one cycle using 1b primitive operations. Memories include the instruction memory of 7 KByte (KB), a line memory of 3 KB, and a frame memory of 165 KB. Instruction memory is used for the 4b PE and stored SIMD Instructions. Line memories are used for previous line data and current line data for the 4b PEs. Frame memory and template are used by the 1b PEs.

The 12b/10b ADC mode is used for conventional viewing without sensing operation. Switching between sensing operation and viewing operation is disabled.

**Figure 12:**
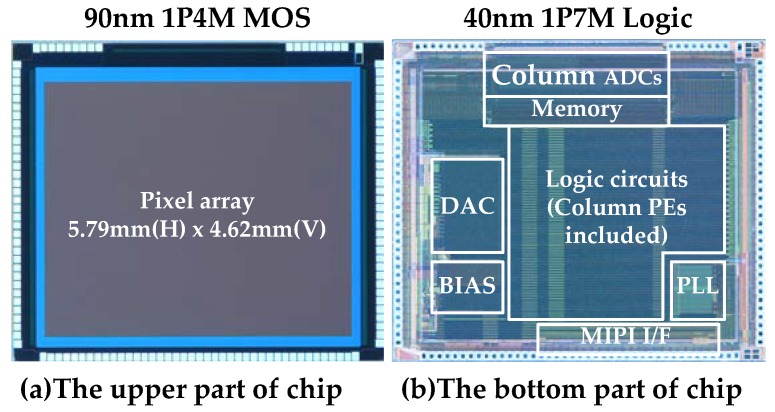
Photomicrograph of the chip. (**a**)The upper chip consists of only pixels; (**b**) the lower chip, using an advanced logic process, contains large amounts of logic.

The chip photomicrograph is shown in Figure 12. The chip size is 9.31 mm (H) × 6.84 mm (V). The pixel array is located in the upper chip. The lower chip contains the column ADCs, memory, and logic circuits including column parallel PEs and MIPI interface.

A performance comparison between three vision chips is summarized in Table 4. From left to right, PE structures are pixel-parallel PEs, matrix-PEs with column ADCs, and this work; two column PEs with column ADC and Memory. This work achieves superior performance in all figures of merit; pixel size, pixel fill factor, array efficiency, processing operations, and power consumption. The solution has high resolution and sensitivity because of the 3D-stacked structure and BI pixel and achieves low power consumption because of the column PE architecture. This results in superior centroid calculation and tracking of faster and smaller objects than conventional vision chips. 

## 4. Experimental Results

### 4.1. Auto Adjusting Function Results

**Figure 13:**
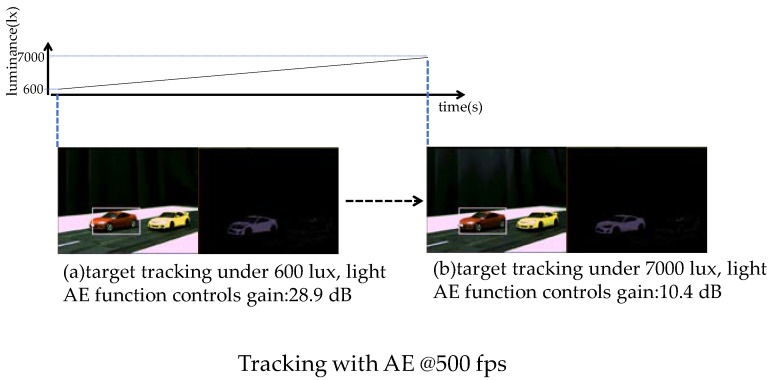
Target tracking with AE/AWB.

The results of imaging and sensing functions are presented in this section. The first result shows the tracking ability of the chip with changes in lighting. The chip performs Auto Exposure (AE) and Automatic White Balance (AWB). The results of target tracking with AE and AWB are shown in Figure 13. This example uses a change in illuminance from 600 lux to 7000 lux. The top of Figure 13 shows a graph of luminance change over time. The horizontal axis is time and the vertical axis is luminance. The left-hand images of (a) and (b) are results of the AE control under different lighting conditions. In this image, the red car is tracked as a target. The right-hand images of (a) and (b) are binarized images. The width and height are configured manually before tracking operations from the external system to enclose the target. AE detects the change in luminance and keeps the image brightness constant by adjusting “shutter time” and “gain” for each frame. Image (a) and image (b) keep the same image brightness by adjusting the gain. Image (a) illustrates tracking an image at 600 lux, whereas image (b) shows tracking an image at 7000 lux. The feedback time for the detection and the controlling is a few frames. Illumination and color temperature change, such as this, can cause target tracking failure because the tracking algorithm uses color information. The AE and AWB functions enable robust target tracking under changing luminance. 

### 4.2. Programmable 4b Column Parallel PEs Spatial Processing Function

The next result shows the capability of the programmable 4b column parallel PEs. The PEs switch between various filters by changing their programming. In high-speed processing, the processor has two problems. First problem is the short-time processing is required, and the Second is that there is very short-time to dynamically change the PE program due to the small instruction memory. The vision chip can solve both problems because the column parallel processing can reduce the number of steps for the spatial processing and the amount of program code for dynamical function switching. An important process in sensing is to reduce noise and make object-detection easier. 

**Figure 14:**
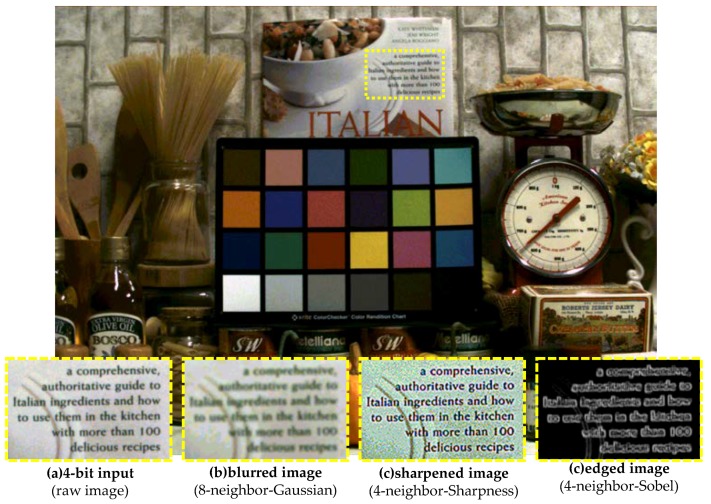
Results of spatial filtering. This is the result of a filter in the yellow area. (**a**) Raw data; (**b**) eight-neighbor Gaussian; (**c**) four-neighbor sharpness; (**d**) four-neighbor Sobel.

The experimental results for spatial processing are shown in Figure 14. There are four images under the larger image, which enhance a highlighted area of text under differing processing within the PEs. Input image (a) is a 4b, 1.27 Mpixel image captured at 500 fps. The four smaller images show the original area of interest and the results of three basic filtering operations: blurring (b), sharpening (c), and edge extraction (d). These basic filters operate correctly for a high-resolution image within only a few cycles. The processing period line by line of all three filters is less than 2 µs, thereby allowing high-speed 500 fps spatial filtering for a 1.27 Mpixel image.

Several spatial filtering algorithms are realized with the programmable ALU. Several example operations and processing step counts are shown in Table 5. The line by line period is a minimum 2 µs and all filters can be implemented in this amount of time. It is possible to execute more complicated filters as well. The number of peripheral pixels used in an eight-neighbor Gaussian filter is twice that of a four-neighbor Gaussian filter. However, the processing can be accomplished with only a slight increase in the number of steps by using the 4b PEs.

**Figure 15:**
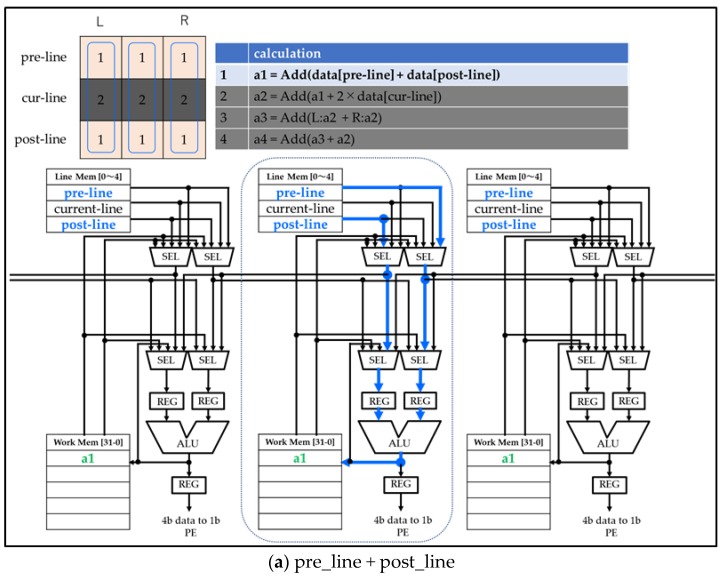

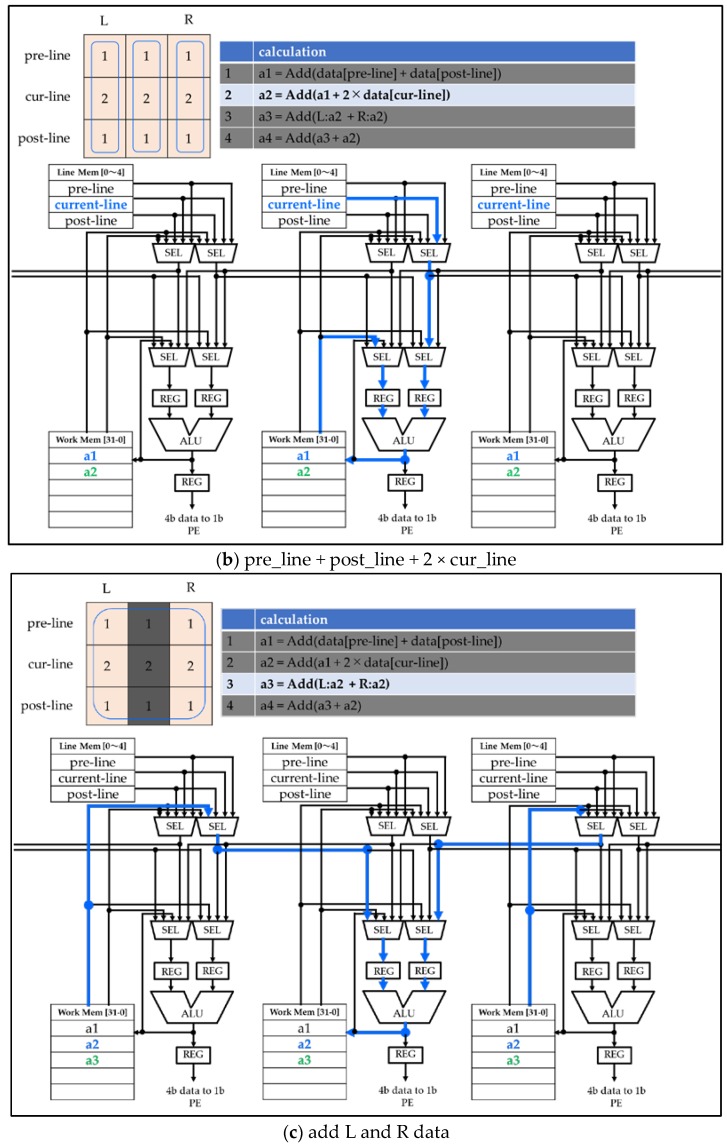

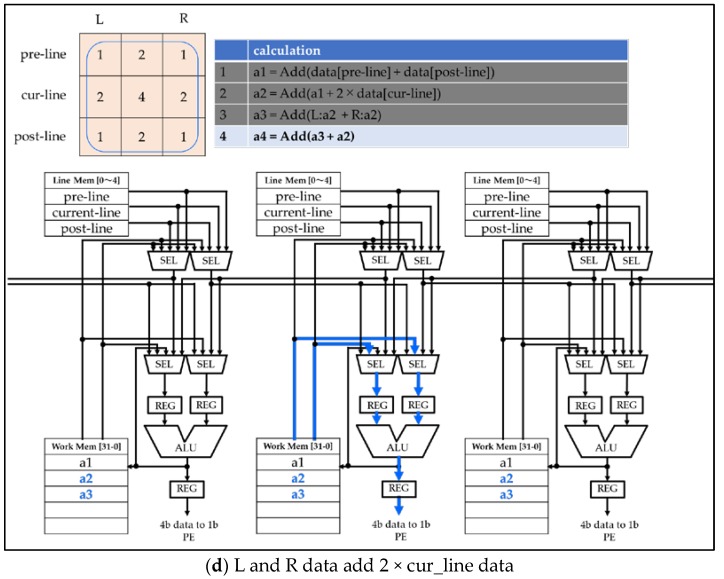
The eight-neighbor Gaussian filter data flow. This is the data flow of ALU step by step. The ALU calculates from (**a**–**d**). Upper-left image is filter tap and coefficient. The filter is using red pixels.

The eight-neighbor Gaussian filter data flow is shown in Figure 15. The eight-neighbor Gaussian is the weighted average of nine pixels. The column parallel PE accomplishes this with four additions. In the figure, the blue data indicate input data and the green data indicate output data. The table in the upper right side shows the calculation of each step. The addition of upper data (pre-line) and lower data (post-line) is shown in Figure 15a. This is followed by the addition of current data ×2, and upper data and lower data are shown in Figure 15b. The PE executes three additions per code word (current pixel and left pixel and right pixel). The addition of left and right data is shown in Figure 15c. The result of filtering is stored in working memory. The addition of left data (L: a2) and right data (R: a2) uses data from the left working memory and the right working memory. The result of the addition is stored in current working memory (a3). The addition of left data and right data is shown in Figure 15d. The PE reduces the number of operational steps by performing orthogonal processing earlier.

**Figure 16:**
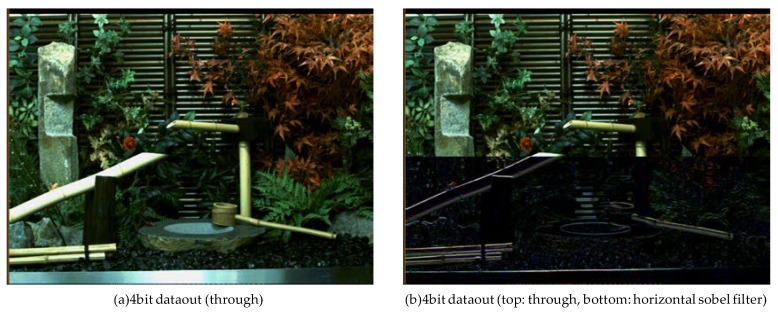
Results of spatial filtering (area selected). (**a**) without filtering; (**b**) with Sobel filtering applied only to the lower half of the image.

The next example shows the result of dynamic programming changes. The result of spatial filtering is shown in Figure 16. Column parallel PEs can hold and select four kinds of programs in the instruction memory. This PE cannot change programs on a per-pixel basis. However, this PE can dynamically switch between these four programs both in time and in screen area. The left side of Figure 16 is the normal output image. In the right image, the upper part of the image is not processed and the lower part of the image is subjected to Sobel filter processing, demonstrating the ability of the system to dynamically switch processing between detection and tracking in the same frame.

## 5. Applications

The vision sensing functions of the vision chip are: (a) 4b spatial processing, (b) target detection and tracking using chroma and temporal information, (c) target feature extraction (moment, centroid, motion vector) using up to six ROIs, and (d) luminance histogram calculation for each color. The vision chip has several functions to improve the accuracy of centroid calculation despite spatiotemporal noise by using 4b noise spatial reduction, 1b foreground extraction, 1b morphological processing, and temporal low pass filtering of the centroid position. There are many applications using these functions for target detection and tracking. Multiple ROIs enable various calculations such as distance and size estimation. Multiple targets with different conditions can be tracked by changing the settings for each ROI. It shows three applications: multi-target tracing, foreground extraction using temporal filtering, and multi-target classification using moments. 

### 5.1. Multi-Target Tracking

**Figure 17:**
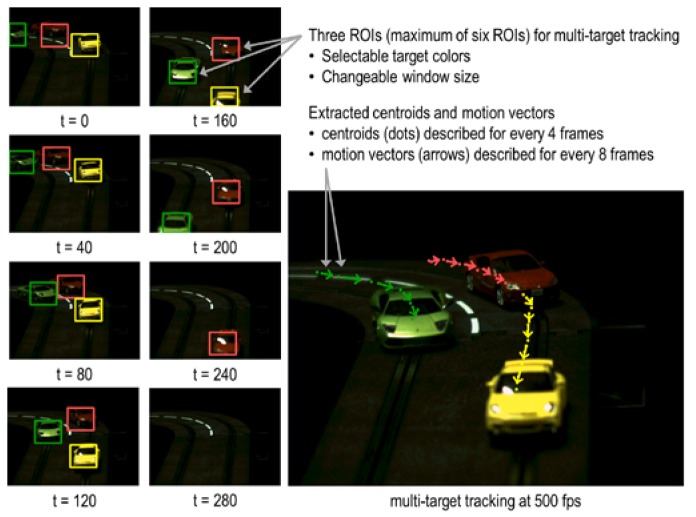
Multi-target tracking. Left-hand images array tracking result time series order and right-hand image shows motion vector and centroid.

The experimental results of multi-target tracking are shown in Figure 17. This demonstrates high-speed multi-target tracking. This is a slot car track with different colored cars. These are the conditions: the frame rate is 500 fps; illuminance is 550 lux under only interior light (incandescent light). The speed of these objects is 2.4 m/s, corresponding to a full-scale car moving at 371 km/h. The left-hand images are arranged in time-series order and each color frame shows a different ROIs. These images show the result of multi-target tracking with three ROIs in each frame. Rectangles show the position, size, and target color of each ROI. The right-hand image shows centroids and motion-vectors as vectors for the three ROI. These are correctly output from the chip as sensing information. Centroids are described for every four frames, and motion vectors are described for every eight frames. High-speed tracking of fast-moving targets is achieved under non-optimal lighting conditions.

### 5.2. Foreground Extraction

The next application is temporal filtering using the 1b PEs and frame memory. The detection method extracts the foreground targets by subtracting the 1b background data stored in the frame memory of the captured image. 

**Figure 18:**
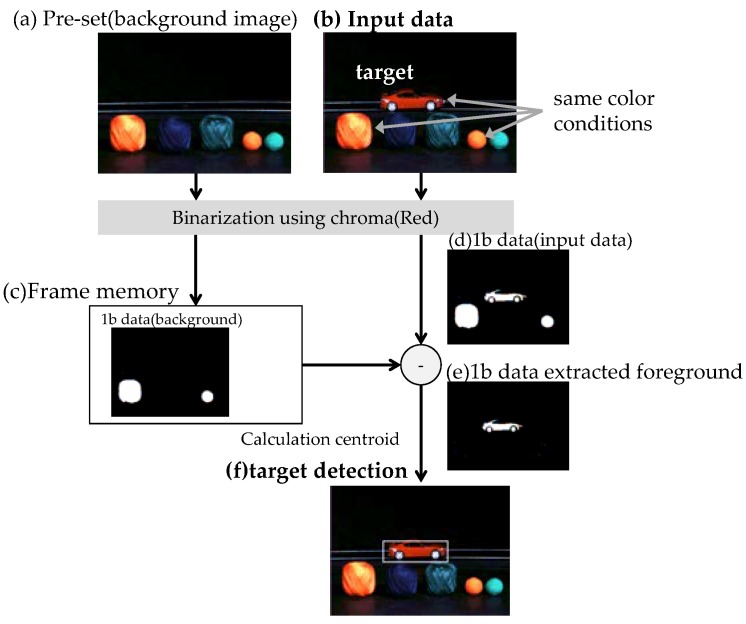
Background difference method using frame memory.

The experimental results of background difference using the frame memory are shown in Figure 18. Even if the same color is contained in the background, the PEs can extract the background by using the frame memory. Image (a) is the background image. Image (b) is the input image. There is a target object of the same color as the object in image (b). The target is the moving car of the same color as the one in the background. In this situation, the chip cannot correctly determine the centroid of the target object. Image (c) is the binarized data of image (a) stored in the frame memory. This operation is executed before detection of the target object. Image (d) is the sorted input image. In detection mode, there is an input image with noise. The background difference is that image (d) is subtracted from image (c). The result of the sorted data is image (e). Using this background difference image (e), the foreground target object is detected and tracked. Although there are some outlier pixels, they do not have a big influence on the calculation of the centroid.

### 5.3. Multi-Target Classification

**Figure 19:**
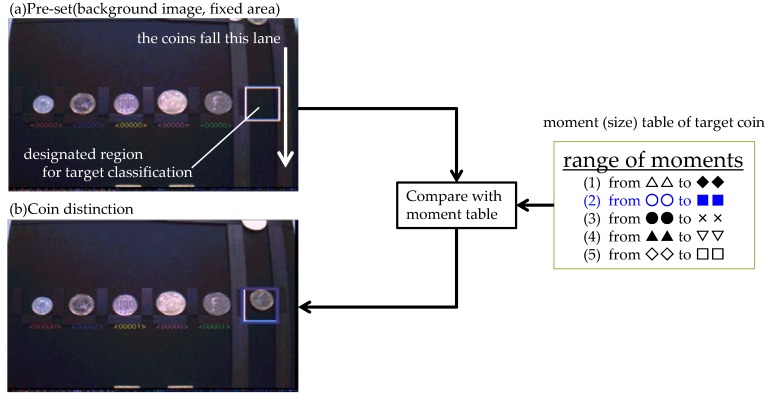
Multi-target classification.

The next application is target classification with multiple windows and target moments. The experimental results of multi-target classification are shown in Figure 19. Each symbol in the right-hand table “range of moments” indicates the value of a zero-order moment. For example, the zero-order moment of one Yen (the coin on the left) is 20. The range can be configured as from 15 to 25 in (1) to classify the coin with some fluctuation of light intensity. This application uses color-sorted information and the target moments to sort coins. There are five types of coins with different sizes in the image (vertical center of image). These five types of coins are target objects to be classified. Coins fall through the right lane in the application system and the application classifies them using stored target moments. This application fixes the position of the detection in five ROIs. Each ROI is selected by a threshold of the moment. Image (a) is awaiting the falling coins. There are counters under each coin type to show the number of fallen coins of each type that have been detected. All counters are zero at first. Image (b) shows the detection of a single coin. The ROIs classify the falling coins using the sensing information (target detect). The vision chip then compares the moment of the detected coin with the stored moment table and classifies the coins by matching the moments. The coin classification is executed when the centroid coordinate of the coin reaches the center coordinate in the Y axis direction of the ROI area. The centroid information is output from the vision chip and the judgment of whether to count the coin is handled by the external processor. The vision chip outputs the results of the classifications as sensing information to an external processor.

## 6. Conclusions

This paper presents a high-speed vision chip with 3D-stacked column parallel ADCs and 140 GOPS programmable SIMD column parallel 4b/1b PEs. The upper chip can achieve high resolution and high sensitivity with back-illuminated pixels and the lower chip, using an advanced logic process, realizes fast and flexible functionality. The vision chip improves the robustness of the imager to lighting variations using high-speed AE and AWB. The 4b PEs realizes highly flexible spatial processing operations. The 4b PEs realizes various filters, each using fewer than 200 cycle steps. These PEs have four kinds of programs and can dynamically switch among them by time or screen area. This dynamic program change function is useful for changing the sensing mode from detection to tracking. The 1b PEs realize highly temporal operations such as noise reduction and extraction. The serial processor calculates target information such as moment, centroid, motion-vector with a maximum of six ROIs, and RGB histogram calculation to process sensing information. Sensing information, such as the moments and centroids, is embedded into the image data and transmitted to the output interface. The external processor can implement various high-speed applications by using sensing information from the vision chip. The chip achieves high speed and functional vision sensing with high sensitivity, high resolution, and high accuracy of target information extraction. 

High-speed multi-target tracking and classification can be applied to factory automation, human interfaces, and gesture control. It can also be applied to a wide variety of sensing applications in real-world situations including general-purpose, low-cost sensors.

## Figures and Tables

**Table 1:** Steps for calculation.

Function	Steps (for Column)
addition	length + 1
subtraction	length + 1
absolute value	length + 1
comparison	length × 2 + 3
select	length + 1
maximum(minimum)value select	length × 2 + 2
logical operation (and/or/xor/xnor/not)	length
swap	length + 3
increment(decrement)	length + 1
move	length

length = bit length of the larger data.

**Table 2:** Functions of the 1b PE.

	Functionality	Data in Frame Memory
Self-Window [21]	Instructed target extraction	Dilated target image
Frame difference	Motion region extraction	Previous image
Background difference method	Foreground target extraction	Background image

**Table 3:** Chip specifications.

Fabrication process		90 nm 1P4M CIS/40 nm 1P7M Logic
Supply voltage		3.3 V/2.9 V/1.8 V/1.1 V
Image size		Diagonal 5.678 mm (Type 1/3.2)
Number of effective pixel		1296 (H) × 976 (V) 1.27 Mpixels
Pixel size		3.50 µm (H) × 3.50 µm (V)
Frame rate	Full	60 fps at 1.27 Mpix 12b
		120 fps at 1.27 Mpix 10b
		500 fps at 1.27 Mpix 4b
	1/2 sub sampling	120 fps at 0.31Mpix 12b
		240 fps at 0.31Mpix 10b
		1000 fps at 0.31 Mpix 4b
Power consumption		230 mW at 1.27 Mpixel 12b 60 fps
		363 mW at 0.31 Mpixel 4b 1000 fps with sensing
		(32 mW at only column PEs)
Saturation signal		19,849 e^−^ at 60 °C
Sensitivity		54,396 e^−^/lx·s
		(Green pixel, 3200 K light source with IR cut filter of 650 nm cut off)
RMS random noise		2.1 e^−^ (AnalogGain:30 dB)
Dynamic range		80 dB at 12b
Maximum operating frequency		108 MHz
Processor count		1 (CP) + 1304 (Column 4b/1bPE)
Instruction cycle		1 cycle
Memory		Instruction memory 7 KByte
		Line memory 3 KByte
		Frame memory 165 Kbyte
		Template memory 12 KByte

**Table 4:** Comparison of vision chips.

Ref. No.	ISSCC 1999 [1]	ISSCC 2014 [2]	This Work
PE Structure	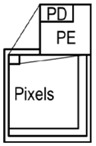	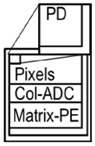	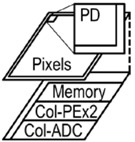
Resolution	16 × 16	256 × 256	1304 × 968
Pixel size	150 μm × 150 μm	10 μm × 10 μm	3.5 μm × 3.5 μm
Pixel fill factor	23%	60%	100%
Chip size	100 mm^2^	82.3 mm^2^	25.7 mm^2^
array efficiency	0.45%	3%	50%
Maximum centroid error ^1^	6.25%	0.39%	0.08%
Power	N/A	630 mW	363 mW
Speed	N/A	108 GOPS	140 GOPS

^1^ Maximum centroid error = 1/(Horizontal Resolution).

**Table 5:** Steps of sample filtering.

Spatial Image Processing	Steps (for Column)	Time (108 MHz)
4-neighbor-Gaussian	21	190 ns
8-neighbor-Gaussian	26	241 ns
4-neighbor-Laplacian	42	389 ns
4-neibor-Sharpness	51	472 ns
4-neighbor-Sobel	79	732 ns
